# Association of magnesium depletion score with serum anti-aging protein Klotho in the middle-aged and older populations

**DOI:** 10.3389/fnut.2025.1518268

**Published:** 2025-03-27

**Authors:** Zhijie Zhuang, Shan Huang, Yingzhu Xiong, Yan Peng, Shuangming Cai

**Affiliations:** ^1^Department of Gastroenterology, Changde Hospital, Xiangya School of Medicine, Central South University, Changde, China; ^2^Department of MICU, Guangdong Women and Children Hospital, Guangzhou, China; ^3^Department of Neurophysiology, Changde Hospital, Xiangya School of Medicine, Central South University, Changde, China; ^4^Department of Internal Medicine, Guangdong Women and Children Hospital, Guangzhou, China

**Keywords:** magnesium depletion score, Klotho, NHANES, nutrition, cross-sectional study

## Abstract

**Background:**

Magnesium deficiency and low levels of the anti-aging protein Klotho have been independently associated with various age-related diseases. The Magnesium Depletion Score (MDS) is recognized as a more valuable and reliable predictor of body magnesium status than traditional clinical markers such as serum and urine magnesium. However, the relationship between magnesium status and serum Klotho levels remains unexplored. This study aimed to investigate the association between magnesium depletion, as quantified by MDS, and serum Klotho levels in US adults.

**Methods:**

We analyzed data from 11,387 participants aged 40–79 years in the National Health and Nutrition Examination Survey (NHANES) 2007–2016. Participants were divided into three groups based on MDS: low (0–1 points), middle (2 points), and high (3–5 points), reflecting cumulative risks of magnesium depletion derived from diuretic use, proton pump inhibitors, renal function, and alcohol intake. Serum Klotho levels were measured using a validated ELISA assay. Sample-weighted multivariable linear regression models were used to examine the association between MDS and serum Klotho levels, adjusting for age, sex, race, socioeconomic status, lifestyle factors (smoking, alcohol use), clinical parameters (body mass index, blood pressure, lipid levels), and energy intake.

**Results:**

The weighted average serum Klotho concentrations decreased significantly across MDS groups (low: 864.50, middle: 805.67, high: 755.02 pg./mL; *p* < 0.0001). After full adjustment, compared to the low MDS group, participants in the middle and high groups had significantly lower serum Klotho levels (*β* = −35.49, 95% CI: −62.29 to −8.69; *β* = −64.82, 95% CI: −115.30 to −14.34, respectively; *p* for trend = 0.003). This inverse association remained consistent across various subgroups, with particularly strong relationships observed in individuals with BMI <25, current smokers, and those with low income.

**Conclusion:**

This study provides novel evidence of an inverse association between MDS, a new valuable indicator of magnesium status, and serum Klotho levels in a large, representative sample of US adults. These findings suggest that monitoring magnesium status via MDS could help identify individuals at risk of accelerated aging, prompting interventions such as dietary adjustments or magnesium supplementation in high-risk populations. Further research is warranted to elucidate the mechanisms underlying this association and its implications for age-related diseases.

## 1 Introduction

Magnesium, the fourth most abundant cation in the human body, plays a crucial role in numerous physiological processes, including energy production, protein synthesis, and the regulation of various enzymes, which highlights its significance in cellular metabolism and overall health (1–4). Despite its importance, magnesium deficiency is a prevalent yet often overlooked condition in the general population, with estimates suggesting that up to 50% of Americans consume less than the recommended daily allowance (5). The consequences of chronic magnesium depletion are far-reaching, affecting multiple organ systems and potentially contributing to the development of various chronic diseases, including cardiovascular disorders, type 2 diabetes, and osteoporosis (6, 7).

Emerging evidence suggests that magnesium status may influence the aging process through multiple mechanisms (8, 9). Inadequate magnesium levels have been linked to increased oxidative stress, inflammation, and mitochondrial dysfunction, all of which are hallmarks of aging (10, 11). These pathways are particularly significant because they overlap with key aging-related processes regulated by anti-aging factors such as Klotho. The relationship between magnesium status and aging is further evidenced by its effects on cellular senescence, telomere length, and age-related disease development (12–14).

Despite the clinical significance of magnesium, accurate assessment of magnesium status remains challenging (15). Traditional markers, such as serum magnesium concentration, often fail to reflect total body magnesium content accurately, as they represent only a small fraction (< 1%) of the body’s magnesium stores (16, 17). To address this limitation, Fan et al. recently developed the Magnesium Depletion Score (MDS), a novel and more comprehensive assessment tool for magnesium status which has been validated against the gold standard magnesium tolerance test (MTT) (18). MDS incorporates multiple factors known to influence magnesium homeostasis, providing a more accurate assessment of magnesium status than traditional markers. Studies have shown that MDS is associated with systemic inflammation and cardiovascular mortality in US adults (18). In comparison to other clinical indicators of magnesium deficiency, MDS has been shown to be more accurate and reliable. Clinically, MDS offers a practical tool to identify individuals with chronic magnesium depletion who may benefit from targeted interventions, potentially mitigating Klotho decline and age-related disease risks.

While the development of MDS has improved our ability to assess magnesium status, understanding its relationship with other aging-related biomarkers is crucial for comprehending the broader role of magnesium in age-related processes. Of particular interest is the anti-aging protein Klotho, which shares several biological pathways with magnesium in regulating aging and metabolism. Klotho has emerged as a critical regulator of aging processes, with significant overlap in the biological pathways affected by magnesium status. Klotho was initially identified as an aging suppressor gene (19). This transmembrane Klotho protein exists in two forms: a membrane-bound form that acts as a co-receptor for fibroblast growth factor 23 (FGF23), and a secreted form that circulates in the blood and cerebrospinal fluid, exerting various systemic effects (20–22). Like magnesium, Klotho plays crucial roles in various physiological processes, including oxidative stress protection and inflammatory regulation. Specifically, serum Klotho exerts various systemic effects, including suppression of insulin/IGF-1 signaling, inhibition of Wnt signaling, and protection against oxidative stress (23–25). Low levels of serum Klotho have been associated with a longevity, cardiovascular diseases, chronic kidney disease, and cognitive decline, making it a potential biomarker of biological aging and overall health status (26–29). The kidney plays a central role in both Klotho expression and magnesium homeostasis. As the primary site of Klotho production, the kidney is responsible for maintaining circulating Klotho levels (30). Simultaneously, it regulates magnesium homeostasis by controlling magnesium reabsorption and excretion (31). This dual role of the kidney makes it particularly important to consider kidney function when examining the relationship between magnesium status and Klotho levels.

Recent research has begun to reveal intriguing connections between magnesium homeostasis and aging-related processes. Evidence from the Wang et al. revealed that each unit increase in MDS was associated with approximately a 30% higher risk for metabolic syndrome, even after adjusting for confounding factors (32). A study by Liu et al. showed that individuals with high MDS had elevated all-cause and CVD death risk (33). Jian et al. provided evidence that elevated MDS was related to a higher abdominal aortic calcification prevalence (34). Given the overlapping roles of magnesium and Klotho in aging processes, particularly in oxidative stress regulation and inflammation, investigating their relationship could provide valuable insights into aging biology. However, the potential association between magnesium depletion, as measured by the MDS, and serum Klotho levels remains unexplored. Intriguingly, emerging evidence suggests a possible link between magnesium status and Klotho expression and function (35, 36). However, the potential association between magnesium depletion, as measured by the MDS, and serum Klotho levels remains unexplored. Based on the emerging evidence and overlapping roles of magnesium and Klotho in aging processes, we hypothesized that magnesium depletion, as measured by MDS, would be associated with lower serum Klotho levels in older adults. Understanding this relationship could provide new insights into aging biology and potential therapeutic targets. Therefore, this study aims to investigate the association between magnesium depletion, as quantified by the Magnesium Depletion Score, and serum anti-aging protein Klotho levels using data from the National Health and Nutrition Examination Survey (NHANES) 2007–2016. By leveraging this large, nationally representative sample of U.S. adults, we seek to provide robust evidence regarding the relationship between these two important factors in aging biology.

## 2 Methods

### 2.1 Study participants

Data for this study were obtained from the National Health and Nutrition Examination Survey (NHANES) database, which covers the period from 2007 to 2016. NHANES is a national survey conducted in the United States to gather information about the noninstitutionalized civilian population. The survey uses a complex, stratified, and multistage probability sampling design to ensure representative results (37). To learn more about the NHANES survey and access detailed information, please visit the official website of the Centers for Disease Control and Prevention at https://www.cdc.gov/nchs/index.htm. The NHANES study protocol has received approval from the National Center for Health Statistics Research Ethics Review Board, and all participants have given written informed consent. The study was conducted in accordance with the Strengthening the Reporting of Observational Studies in Epidemiology guidelines, which provide a framework for reporting cross-sectional studies (38).

**Figure 1:**
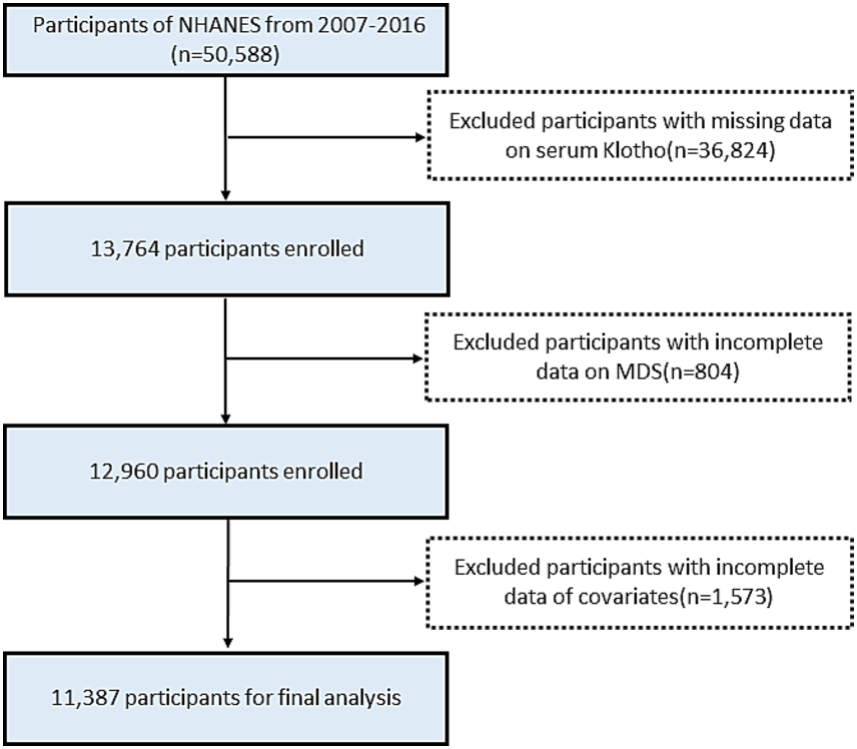
Flowchart of the participant selection from NHANES 2007–2016.

The survey was limited to individuals in the United States who were middle-aged and older, specifically those aged 40–79 years. Serum klotho levels were not measured in participants aged<40 years. After excluding participants with missing serum klotho data (*n* = 36,824), participants with incomplete data on MDS (*n* = 804), and participants with incomplete data of covariates (*n* = 1,573), a total of 11,387 participants were included in the final analysis (Figure 1).

### 2.2 Measurement of serum Klotho concentrations

The original frozen serum samples used to measure serum Klotho of participants aged 40–79 years, collected during the NHANES 2007–2016 cycles, were stored at a temperature of −80°*C. prior* to the study commencement, an extensive validation of the ELISA method was conducted for measuring Klotho concentration in human samples. The Klotho concentration analysis was performed by the Northwest Lipid Metabolism and Diabetes Research Laboratory at the University of Washington using a commercially available ELISA kit (IBL International Corporation, Gunma, Japan). All samples were tested in duplicate, and the average of the two measurements was considered the final Klotho concentration. Samples with a difference of more than 10% between duplicate values were re-measured. The quality control measures included validation of standard curves against manufacturer criteria, and evaluation of assay linearity (R^2^ values >0.997). The intra-assay precision showed CV values of 2.3–3.3% for human samples, while the inter-assay precision yielded CV values of 3.4–3.8%. Quality control samples with values exceeding two standard deviations from the established value were considered invalid for the entire plate and retested. The sensitivity of the Klotho concentration assay was 4.33 pg./mL. To establish a reference range, a set of 114 samples from healthy donors was evaluated, yielding a range of 285.8 to 1638.6 pg./mL with a mean concentration of 698.0 pg./mL. For a more detailed description of the process of testing serum Klotho concentrations, please refer to the NHANES website.^1^

### 2.3 Assessment of magnesium depletion score (MDS)

#### 2.3.1 Components and calculation of MDS

MDS is a clinical composite index devised to assess magnesium deficiency within the body. For this study, we calculated MDS specifically using data from the NHANES database (2007–2016), following the established algorithm that evaluates four key risk factors:

1. Diuretic use (current use: 1 point)
2. Proton pump inhibitor (PPI) use (current use: 1 point)
3. Kidney function:

- 60 mL/min/1.73 m^2^ ≤ eGFR <90 mL/(min/1.73 m^2^): 1 point.
- eGFR <60 mL/(min/1.73 m^2^): 2 points.

1. Alcohol consumption (heavy drinker: 1 point; defined as >1 drink/day for women and > 2 drinks/day for men)

#### 2.3.2 MDS classification

Based on the total points calculated from these components, participants were stratified into three groups:

- Low MDS: 0–1 points.
- Middle MDS: 2 points.
- High MDS: 3–5 points.

### 2.4 Covariates

Potential covariates were selected based on numerous influencing factors listed in previous literature and clinical expertise (34). The demographic characteristics include age, sex (male and female), income poverty ratio (PIR), marital status, education level (below high school, High school and above high school), and race (non-Hispanic White, Mexican Americans, non-Hispanic Black, other Hispanic and or other Race). Dietary data were collected using two 24-h dietary recalls conducted by trained interviewers. Energy intake was calculated by averaging the total caloric intake from these two 24-h recalls, using the USDA Food and Nutrient Database for Dietary Studies (FNDDS) versions corresponding to each NHANES cycle (2007–2016). For participants with only one valid recall day, that single value was used. The average energy intake was expressed as kilocalories per day (kcal/day) in our analyses. The dietary magnesium intake value was determined by averaging the 2 sets of 24-h recall data; The physical examination data include body mass index (BMI), waist circumference (WC), systolic blood pressure (SBP), and diastolic blood pressure (DBP); The laboratory test results include total cholesterol (TC), total triglyceride (TG), high-density lipoprotein (HDL) cholesterol, and calcium level; The lifestyle behaviors: smoking status and drinking status; Comorbidities were defined based on standard clinical criteria: diabetes mellitus (self-reported diagnosis, use of diabetes medications, or laboratory evidence of hyperglycemia), hypertension (self-reported diagnosis, use of anti-hypertensive medications, or elevated blood pressure), and hyperlipidemia (self-reported diagnosis or abnormal lipid profile according to standard clinical thresholds).

### 2.5 Statistical analysis

The data were processed in accordance with NHANES analytical guidelines. All analyses used appropriate sample weights and strata because of the complex sampling design of NHANES. Continuous variables were summarized as the weighted mean ± standard error and examined using Student t test or one-way ANOVA. Categorical variables were reported as number and weighted percentages, and comparison between groups was investigated using the Chi-square test. In our study, participants with missing data on key variables were excluded from the analysis. No multiple imputation or other methods were used.

We applied sample-weighted multivariable linear regression models to evaluate the relationship between the MDS and serum Klotho concentrations. Model 1 was adjusted for nothing. Model 2 was adjusted for age, sex, and race. Model 3 was based on model 2 and covariates including education level, marital status, PIR, smoking status, drinking status, BMI, WC, SBP, DBP. Model 4 was based on model 3 and covariates including TG, TC, HDL, energy intake. Subgroup analyses stratified by age, sex, BMI, education level, smoking status, drinking status, PIR, hypertension, and diabetes were conducted using stratified multivariate regression analysis. These specific subgroups were selected based on prior evidence of effect modification: (1) age and sex are known to influence both Klotho expression and magnesium metabolism; (2) BMI and lifestyle factors (smoking and drinking) may modify mineral homeostasis and Klotho levels; (3) socioeconomic factors (education level and PIR) might affect dietary patterns and medication use that impact magnesium status; and (4) chronic conditions like hypertension and diabetes have been associated with altered Klotho and magnesium levels. In addition, *p* values for interaction across subgroups were also calculated by the likelihood ratio test.

All analyses in this study were conducted utilizing R (version 4.3.3). Values of *p* < 0.05 on both sides were considered to be statistically significant.

## 3 Results

### 3.1 Characteristics of study participants

**Figure 2:**
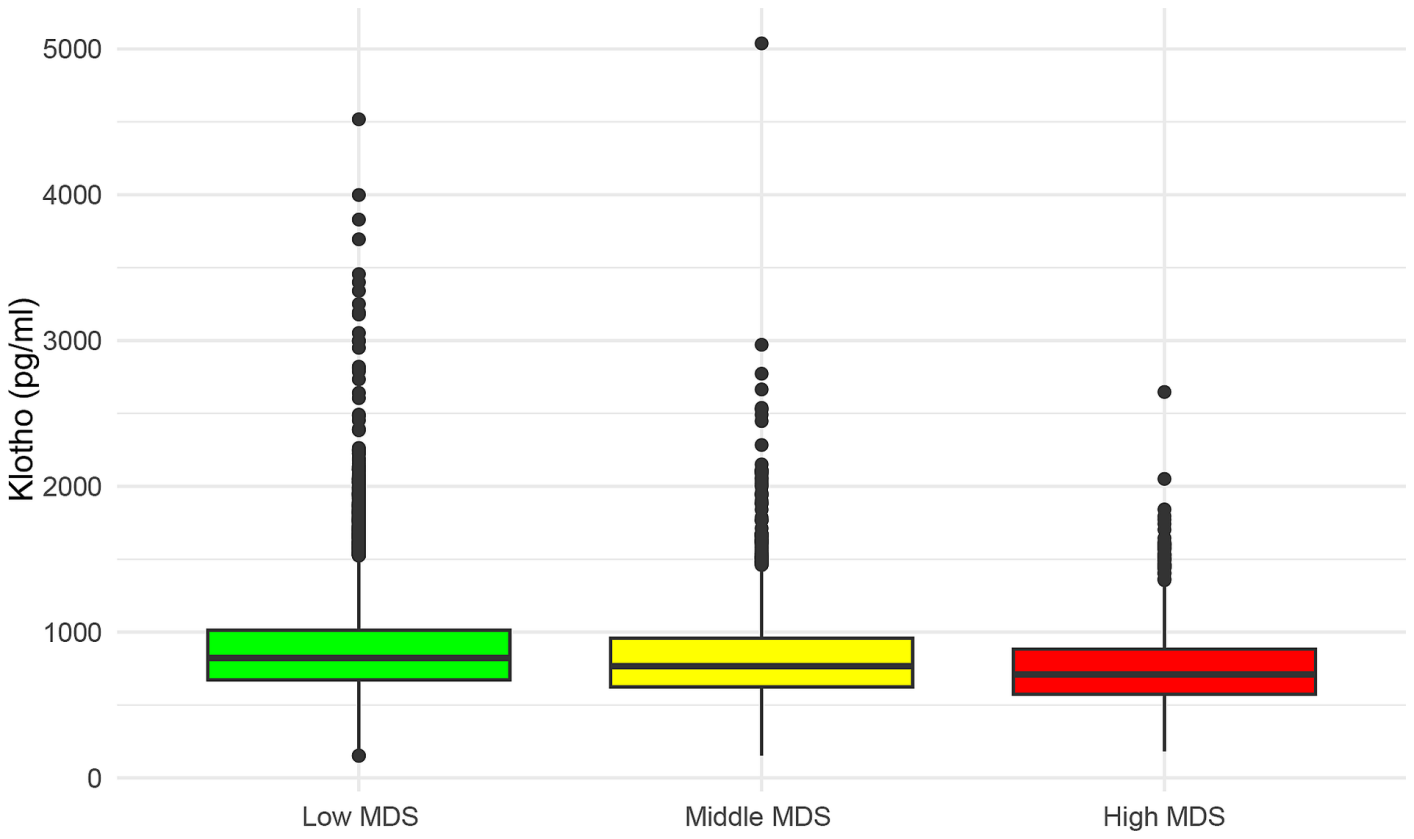
The distribution of serum Klotho levels in different MDS groups. MDS, magnesium depletion score.

As illustrated in Table 1, baseline characteristics of participants are presented according to three Magnesium Depletion Score (MDS) groups: High MDS (*n* = 1,079), Middle MDS (*n* = 2,326), and Low MDS (*n* = 7,982). A total of 11,387 participants were included in the study. The mean age of all participants was 56.25 ± 0.16 years and 5,596 (weighted percentage, 48.13%) were men. The weighted average serum Klotho concentrations in the MDS (High to Low) groups were 755.02 pg./mL, 805.67 pg./mL, and 864.50 pg./mL, respectively, and the differences between the groups were statistically significant (*p* < 0.0001). In addition, as the MDS increased, the serum Klotho levels tended to decrease (Figure 2).

**Table 1:** Characteristics of the study participants by MDS groups.

Characteristics	Overall (*N* = 11,387)	Low MDS (*N* = 7,982)	Middle MDS (*N* = 2,326)	High MDS (*N* = 1,079)	*p* value
Age (years)	56.25 (0.16)	53.89 (0.17)	60.78 (0.31)	65.13 (0.47)	< 0.0001
Age group (%)					< 0.0001
40–49	3,121 (31.25)	2,770 (38.59)	292 (16.32)	59(5.75)	
50–59	3,004 (31.29)	2,341 (33.32)	498 (28.19)	165 (21.74)	
60–69	3,230 (24.16)	2033 (20.72)	824 (31.96)	373 (34.20)	
70–79	2032 (13.30)	838(7.37)	712 (23.53)	482 (38.31)	
Sex (%)					< 0.0001
Female	5,791 (51.87)	4,031 (50.87)	1,158 (51.19)	602 (61.85)	
Male	5,596 (48.13)	3,951 (49.13)	1,168 (48.81)	477 (38.15)	
Race (%)					< 0.0001
Non-Hispanic White	5,255 (75.38)	3,389 (72.84)	1,259 (81.55)	607 (81.76)	
Mexican American	1741 (6.13)	1,397 (7.29)	254 (3.55)	90 (2.66)	
Non-Hispanic Black	2,254 (8.79)	1,502(8.71)	488(8.42)	264 (10.36)	
Other Hispanic	1,209 (4.12)	937 (4.76)	195 (2.61)	77 (2.41)	
Other Race	928 (5.58)	757 (6.40)	130 (3.87)	41 (2.81)	
Marital status (%)					< 0.0001
Divorced	1751 (13.94)	1,214 (13.83)	361 (13.79)	176 (15.24)	
Living with partner	543 (4.50)	411 (4.70)	102 (4.43)	30 (2.93)	
Married	6,803 (65.95)	4,858 (66.79)	1,342 (64.13)	603 (63.31)	
Never married	930 (7.19)	691 (7.62)	176 (7.10)	63 (3.79)	
Separated	432 (2.48)	315 (2.49)	85 (2.71)	32 (1.89)	
Widowed	928 (5.93)	493(4.57)	260(7.83)	175 (12.84)	
Education level (%)					0.04
Below high school	2,987 (15.36)	2,121 (15.60)	570 (13.47)	296 (17.95)	
High school	2,541 (22.25)	1732 (22.04)	542 (22.76)	267 (22.83)	
Above high school	5,853 (62.37)	4,124 (62.36)	1,214 (63.77)	515 (59.22)	
PIR (%)					< 0.001
<1.3	3,402 (17.13)	2,382 (17.24)	658 (15.15)	362 (20.99)	
1.3–3.5	4,124 (32.92)	2,871 (32.33)	844 (33.46)	409 (36.58)	
>3.5	3,861 (49.96)	2,729 (50.44)	824 (51.39)	308 (42.43)	
Smoking status (%)					< 0.0001
Never	5,735 (51.32)	4,207 (53.12)	1,064 (47.23)	464 (46.31)	
Former	3,423 (30.53)	2,116 (27.31)	850 (37.06)	457 (41.96)	
Now	2,225 (18.12)	1,655 (19.58)	412 (15.71)	158 (11.73)	
Drinking status (%)					0.002
Never	1,570 (9.98)	1,123 (10.24)	288(8.57)	159 (11.16)	
Former	2,506 (18.12)	1,676 (17.67)	525 (17.84)	305 (22.59)	
Now	7,311 (71.90)	5,183 (72.08)	1,513 (73.59)	615 (66.26)	
BMI (kg/m^2^)	29.63 (0.11)	29.24 (0.12)	30.19 (0.19)	31.57 (0.33)	< 0.0001
BMI group (%)					< 0.0001
< 25	2,600 (23.99)	1991 (25.84)	451 (21.19)	158 (16.94)	
25–30	3,903 (34.79)	2,824 (35.56)	763 (34.31)	316 (31.98)	
> 30	4,782 (40.59)	3,114 (38.59)	1,084 (44.51)	584 (51.08)	
WC (cm)	102.03 (0.26)	100.87 (0.30)	104.18 (0.43)	106.74 (0.62)	< 0.0001
SBP (mmHg)	124.33 (0.26)	123.05 (0.31)	127.06 (0.44)	128.49 (0.64)	< 0.0001
DBP (mmHg)	71.90 (0.24)	72.51 (0.26)	71.34 (0.37)	68.04 (0.55)	< 0.0001
TG (mmol/L)	1.53 (0.02)	1.50 (0.03)	1.58 (0.05)	1.65 (0.07)	0.04
TC (mmol/L)	5.21 (0.02)	5.24 (0.02)	5.17 (0.03)	5.01 (0.06)	< 0.0001
HDL cholesterol (mmol/L)	1.40 (0.01)	1.38 (0.01)	1.44 (0.02)	1.43 (0.02)	< 0.0001
Energy intake (kcal/day)	2047.93 (11.40)	2079.74 (13.18)	2023.82 (23.48)	1842.32 (32.38)	< 0.0001
Magnesium intake (mg/day)	306.77 (2.56)	312.69 (2.65)	301.77 (4.61)	269.79 (4.33)	< 0.0001
Calcium (mg/dL)	9.40 (0.01)	9.38 (0.01)	9.46 (0.01)	9.46 (0.02)	< 0.0001
Hypertension (%)					< 0.0001
No	5,191 (51.24)	4,451 (60.49)	626 (34.36)	114 (14.50)	
Yes	6,195 (48.75)	3,530 (39.51)	1700 (65.64)	965 (85.50)	
DM (%)					< 0.0001
No	7,241 (69.93)	5,419 (73.31)	1,328 (65.08)	494 (53.73)	
Yes	4,139 (30.02)	2,556 (26.69)	998 (34.92)	585 (46.27)	
Hyperlipidemia (%)					< 0.0001
No	2,245 (19.84)	1753 (22.55)	376 (14.76)	116(9.41)	
Yes	9,142 (80.16)	6,229 (77.45)	1950 (85.24)	963 (90.59)	
Klotho(pg/mL)	843.19 (5.38)	864.50(5.71)	805.67(9.05)	755.02 (12.52)	< 0.0001
Klotho group (%)					< 0.0001
Q1	2,848 (24.69)	1749 (21.90)	677 (29.34)	422 (36.92)	
Q2	2,847 (26.12)	1967 (25.58)	602 (26.92)	278 (28.77)	
Q3	2,845 (25.61)	2099 (26.58)	548 (24.80)	198 (19.41)	
Q4	2,847 (23.57)	2,167 (25.94)	499 (18.94)	181 (14.90)	

MDS, magnesium depletion score; PIR, family poverty income ratio; BMI, body mass index; WC, waist circumference; SBP, systolic blood pressure; DBP, diastolic blood pressure; TG, total triglyceride; TC, total cholesterol; HDL, high-density lipoprotein cholesterol; DM, diabetes mellitus. Categorical variables are presented as numbers (percentages). Sampling weights were applied for calculation of demographic descriptive statistics. N reflect the study sample while percentages reflect the survey-weighted data.

Participants in the High MDS group were older, with a mean age of 65.13 years, compared to those in the Middle and Low MDS groups (60.78 and 53.89 years, respectively; *p* < 0.0001). The High MDS group also had a higher percentage of females (61.85%) compared to the Middle (51.19%) and Low MDS groups (50.87%; *p* < 0.0001). In terms of race, the High MDS group had a greater proportion of non-Hispanic White (81.76%) compared to the Middle and Low MDS groups (81.55 and 72.84%, respectively; *p* < 0.0001). Additionally, participants with higher MDS had higher levels of triglycerides (TG). The prevalence of hypertension, diabetes mellitus (DM), and hyperlipidemia increased with higher MDS.

### 3.2 Association between MDS and serum Klotho

Weighted multiple regression analysis was employed to examine the relationship between MDS and serum klotho levels (Table 2). When analyzed as a continuous variable, MDS showed a significant inverse association with serum klotho levels across all models, with the fully adjusted model showing *β* = −23.16 (95%CI: −36.98 to −9.34).

**Table 2:** Association between MDS and serum Klotho.

	β (95% CI)			
	Model 1	Model 2	Model 3	Model 4
MDS	−40.56(−48.18,-32.93)	−37.32(−45.46,-29.19)	−34.34(−42.90,-25.77)	−23.16(−36.98, −9.34)
MDS group				
Low	Reference	Reference	Reference	Reference
Middle	−58.83(−77.61,-40.04)	−50.77(−71.19,-30.35)	−45.79(−66.47,-25.12)	−35.49(−62.29, −8.69)
High	−109.48(−134.21,-84.76)	−102.45(−128.07,-76.84)	−98.81(−125.03,-72.60)	−64.82(−115.30,-14.34)
*p* for trend	<0.0001	<0.0001	<0.0001	0.003

CI, confidence interval; MDS, magnesium depletion score; PIR, family poverty income ratio; BMI, body mass index; WC, waist circumference; SBP, systolic blood pressure; DBP, diastolic blood pressure; TG, total triglyceride; TC, total cholesterol; HDL, high-density lipoprotein cholesterol. Model 1: unadjusted. Model 2: adjusted for age, sex, race. Model 3: adjusted for all the factors in Model 2 and education, marital status, PIR, smoking status, drinking status, BMI, WC, SBP, DBP. Model 4: adjusted for all the factors in Model 3 and TG, TC, HDL, energy intake.

When MDS was categorized, compared to the low group, both middle and high MDS groups demonstrated progressively lower serum klotho levels. In the fully adjusted model (Model 4), participants in the middle MDS group showed a 35.49 pg./mL decrease (95%CI: −62.29 to −8.69), while those in the high MDS group showed a more substantial decrease of 64.82 pg./mL (95%CI: −115.30 to −14.34) in serum Klotho levels compared to the low MDS group. This dose-dependent relationship remained significant after adjusting for all covariates (p for trend = 0.003).

To examine whether the observed association between MDS and serum Klotho levels was independent of calcium status, we conducted a sensitivity analysis (Supplementary Table S1). Both continuous (*β* = −41.59, 95%CI: −49.25 to −33.92) and categorical analyses (middle vs. low: *β* = −60.61, 95%CI: −79.44 to −41.77; high vs. low: β = −111.26, 95%CI: −135.83 to −86.69; p for trend <0.0001) demonstrated that this association still remained with adjustment for calcium levels. Additional sensitivity analyses using an alternative MDS grouping method (MDS < 3 vs. MDS ≥ 3), showed consistent results (Supplementary Table S2). After full adjustment, participants with MDS ≥ 3 had significantly lower serum Klotho levels compared to those with MDS < 3 (*β* = −52.6, 95%CI: −101.02 to −4.19; *p* = 0.03). To address potential confounding by magnesium intake, we conducted additional sensitivity analyses (Supplementary Table S3). After adjusting for magnesium intake, the inverse association between MDS and serum Klotho levels remained significant (middle vs. low: *β* = −59.02, 95%CI: −77.09 to −40.96; high vs. low: β = −109.66, 95%CI: −135.17 to −84.15; p for trend <0.0001).

### 3.3 Subgroup analysis

Subgroup analysis were performed to examine the robustness of the association between MDS and serum klotho levels in different population settings, including age, sex, BMI, education level, smoking status, drinking status, PIR, hypertension, and diabetes (Table 3). Additionally, interaction tests were conducted among different subgroups.

**Table 3:** Subgroup analysis for the association between MDS and serum Klotho.

	Magnesium depletion score, β(95% CI)			P for trend	P for interaction
	Low	Middle	High		
Age					0.5
40–49	Ref	−82.373(−140.102, −24.644)	−6.59(−219.790, 206.609)	0.071	
50–59	Ref	−42.237(−88.647, 4.173)	−109.762(−185.674, −33.850)	0.001	
60–69	Ref	−4.286(−62.482, 53.910)	−49.139(−137.711, 39.433)	0.351	
70–79	Ref	−27.947(−78.936, 23.042)	−43.536(−107.248, 20.177)	0.147	
Sex					0.976
Female	Ref	−32.866(−70.718, 4.985)	−62.951(−112.025, −13.876)	0.007	
Male	Ref	−40.78(−77.313, −4.246)	−61.357(−138.343, 15.629)	0.035	
BMI					0.053
< 25	Ref	−88.985(−151.974, −25.996)	−171.987(−273.356, −70.618)	<0.001	
25–30	Ref	−1.902(−58.035, 54.232)	−90.378(−155.498, −25.257)	0.057	
> 30	Ref	−35.852(−73.879, 2.176)	−12.161(−88.619, 64.297)	0.375	
Education level					0.845
Below high school	Ref	−16.697(−86.805, 53.411)	−96.302(−183.340, −9.264)	0.046	
High school	Ref	−44.314(−100.175, 11.547)	−69.566(−147.748, 8.616)	0.044	
Above high school	Ref	−36.403(−71.349, −1.458)	−49.283(−113.073, 14.508)	0.025	
Smoking status					0.205
Never	Ref	−33.514(−72.199, 5.171)	−49.447(−105.743, 6.848)	0.033	
Former	Ref	−18.42(−69.227, 32.388)	−40.736(−110.914, 29.443)	0.245	
Now	Ref	−84.36(−137.889, −30.830)	−234.28(−333.564, −134.996)	<0.0001	
Drinking status					0.308
Never	Ref	81.878(−14.407, 178.164)	−10.287(−129.309, 108.736)	0.473	
Former	Ref	−30.711(−94.549, 33.128)	−99.765(−187.908, -11.622)	0.028	
Now	Ref	−48.17(−81.109,-15.230)	−57.093(−118.814, 4.629)	0.007	
PIR					0.081
<1.3	Ref	−10.363(−66.156, 45.430)	−135.94(−206.433, −65.446)	0.002	
1.3–3.5	Ref	−51.076(−98.176, −3.976)	−104.915(−171.814, −38.016)	0.002	
>3.5	Ref	−32.631(−70.410, 5.148)	−5.362(−82.738, 72.013)	0.392	
Hypertension					0.779
No	Ref	−16.36(−72.239, 39.519)	−63.913(−153.968, 26.142)	0.272	
Yes	Ref	−48.265(−76.461,-20.070)	−73.643(−128.844, −18.442)	0.002	
DM					0.867
No	Ref	−38.043(−66.556, −9.530)	−68.927(−145.156, 7.302)	0.014	
Yes	Ref	−39.701(−77.901, −1.501)	−69.135(−124.373, −13.896)	0.005	

Each stratification was adjusted for age, sex, race, education, marital status, PIR, smoking status, drinking status, BMI, WC, SBP, DBP, TG, TC, HDL, and energy intake except the stratification factor itself. CI, confidence interval; MDS, magnesium depletion score; PIR, family poverty income ratio; BMI, body mass index; WC, waist circumference; SBP, systolic blood pressure; DBP, diastolic blood pressure; TG, total triglyceride; TC, total cholesterol; HDL, high-density lipoprotein cholesterol.

The results of fully adjusted β coefficients across different subgroups were mostly consistent with the main findings, indicating that participants with higher MDS had lower serum klotho levels. Statistically significant negative associations were observed in several subgroups (p for trend <0.05), including age group 50–59, both sexes, BMI <25, all education levels, never and current smokers, former and current drinkers, low and middle PIR, and both hypertension and diabetes status. The most substantial reductions in serum Klotho levels were observed in three key subgroups when comparing high versus low MDS groups: current smokers demonstrated the largest decrease of 234.28 pg./mL (95%CI: −333.564 to −134.996; p for trend <0.0001), followed by individuals with BMI <25 showing a 171.99 pg./mL decrease (95%CI: −273.356 to −70.618; p for trend <0.001), and those with low PIR exhibiting a 135.94 pg./mL decrease (95%CI: −206.433 to −65.446; p for trend =0.002). As for interaction terms, no variables significantly affected the relationship between MDS and serum klotho levels (all *p* for interaction >0.05). The closest to significance was BMI (*p* for interaction = 0.053), suggesting a potential difference in the association across BMI categories.

These subgroup analyses generally support the main findings of a negative association between MDS and serum *α*-klotho levels, with some variations in the strength of the association across different population characteristics.

## 4 Discussion

This study aimed to investigate the association between the Magnesium Depletion Score (MDS) and serum levels of the anti-aging protein Klotho in U.S. adults. Our analysis of data from 11,387 participants aged 40–79 years in the National Health and Nutrition Examination Survey (NHANES) 2007–2016 revealed a statistically significant inverse relationship between MDS and serum Klotho levels, which persisted across different statistical models and population subgroups. Notably, participants with higher MDS had significantly lower serum Klotho concentrations, with the most substantial reductions observed in those in the high MDS group. Our results suggest that magnesium depletion may contribute to the age-related decline in Klotho levels, an anti-aging protein.

The magnitude of Klotho reduction observed in our study has important clinical implications. Previous research has established that decreases in serum Klotho levels are associated with various adverse health outcomes. Research has demonstrated that individuals with low Klotho levels have more than two-fold increased risk of cardiovascular mortality (39), and a 76% higher risk of cardiovascular events (40). In terms of mortality, low Klotho levels were associated with a 21% increase in all-cause mortality risk (40). Therefore, the 64.82 pg./mL average decrease we observed in the high MDS group suggests potentially significant health implications. In our study, the substantial reductions in serum Klotho levels were particularly notable in specific subgroups, with the most pronounced decreases observed in current smokers (234.28 pg./mL) and individuals with BMI <25 (171.99 pg./mL). These findings suggest that certain population subgroups may be more vulnerable to the effects of magnesium depletion on Klotho levels, highlighting the importance of maintaining adequate magnesium status, particularly in these potentially at-risk populations.

Numerous studies have established the crucial role of magnesium in various physiological processes and its association with aging-related diseases. Magnesium is essential for mitochondrial function, which plays a key role in cellular energy production and longevity (41). Mitochondrial dysfunction is a hallmark of aging, often associated with increased production of reactive oxygen species (ROS) and oxidative stress (42, 43). Our findings of a significant inverse association between MDS and serum Klotho levels suggest that magnesium depletion might compromise the body’s anti-aging mechanisms, particularly through its interaction with Klotho. Similar to Klotho, which has been shown to protect against oxidative stress and regulate mitochondrial function (44), magnesium deficiency has been shown to accelerate the aging process by impairing mitochondrial function and increasing ROS, which damages cellular components, including lipids, proteins, and DNA (9, 45). A study by Barbagallo et al. (46) emphasized that magnesium deficiency leads to oxidative stress, which not only accelerates cellular senescence but also contributes to the development of age-related diseases such as cardiovascular disease, neurodegenerative disorders, and type 2 diabetes. This parallel with Klotho’s protective effects against age-related diseases highlights the potential synergistic relationship between these two factors in preventing age-related oxidative damage (47). In animal studies, magnesium supplementation has been shown to extend lifespan by improving mitochondrial health and reducing oxidative damage, further supporting the link between magnesium and aging (48).

Chronic magnesium deficiency is a common issue, particularly among older adults, where magnesium intake often falls below the recommended levels (9). This aligns with our observation that participants in the High MDS group were significantly older (mean age 65.13 years) compared to those in the Middle and Low MDS groups. Studies have found that magnesium deficiency is associated with several biomarkers of aging, including telomere shortening (49) and systemic inflammation (18). Like Klotho, which has been shown to regulate inflammatory pathways and maintain telomere integrity (50), magnesium has been suggested to play a role in maintaining telomere length by reducing oxidative stress and inflammation. In a population-based study, a significant correlation was observed between low magnesium levels and shorter telomeres, suggesting that magnesium deficiency may accelerate biological aging (51). Furthermore, magnesium deficiency has been linked to increased levels of pro-inflammatory cytokines, such as interleukin-6 (IL-6) and tumor necrosis factor-alpha (TNF-*α*), both of which are associated with the aging process (52). These inflammatory markers are also known to be regulated by Klotho, suggesting a potential mechanistic link between magnesium status and Klotho function in modulating inflammation (53). Chronic inflammation, often referred to as “inflammaging, “is a well-known contributor to age-related diseases, and both magnesium and Klotho’s anti-inflammatory properties are thought to work in concert to counteract this effect (54). A clinical study by King et al. (55) demonstrated that magnesium supplementation reduced inflammatory markers in elderly individuals, thereby suggesting that these two factors may work synergistically in protecting against inflammaging.

MDS, the newly developed assessment metric of magnesium status, integrates 4 key risk factors that influence magnesium reabsorption: alcohol consumption, diuretic use, proton pump inhibitor (PPI) use, and kidney function (18). Alcohol acts as a magnesium diuretic by rapidly increasing magnesium excretion through proximal renal tubular dysfunction, which can occur even in individuals with a negative magnesium balance (56). Diuretics, such as furosemide, reduce the positive transepithelial membrane potential in the thick ascending limb (TAL) of the loop of Henle, leading to magnesium wasting (57, 58). PPIs, including omeprazole, interfere with intestinal magnesium absorption by elevating the pH in the small intestine and downregulating TRPM6 activity, with higher PPI doses exacerbating this effect (59, 60). The kidneys play a critical role in maintaining magnesium homeostasis, reabsorbing over 80% of serum magnesium (2); however, patients with chronic kidney disease (CKD) experience increased urinary magnesium excretion, which can result in hypomagnesemia. Heavy alcohol consumption further contributes to magnesium depletion by increasing urinary magnesium and calcium excretion. Chronic magnesium deficiency, driven by these factors, is associated with the progression of cardiovascular diseases, such as atherosclerosis (1). Given the crucial role of kidney function in magnesium homeostasis, it is important to note that our study included participants with varying degrees of kidney function, as assessed through eGFR levels in the MDS calculation. The relationship between magnesium status and Klotho levels should be considered within the context of kidney function. Chronic kidney disease (CKD) is known to affect both Klotho expression and magnesium homeostasis. The kidney is a major site of Klotho production, and CKD is associated with reduced Klotho levels (30). Similarly, CKD can impact magnesium handling, potentially contributing to magnesium dysregulation (31). Rather than excluding participants with CKD, we incorporated kidney function into our analysis through the MDS scoring system, which assigns points based on eGFR levels. This approach allowed us to examine the relationship between magnesium status and Klotho levels while accounting for the influence of kidney function. However, future studies may benefit from specifically analyzing this relationship in different CKD stages to better understand how varying degrees of kidney dysfunction might modify the association between magnesium status and Klotho levels.

To our knowledge, this is the first study to employ MDS as a novel indicator of magnesium status in relation to serum Klotho levels in a representative population of US adults. Our findings of lower serum Klotho levels in individuals with higher MDS (progressively decreasing from 864.50 pg./mL in low MDS to 755.02 pg./mL in high MDS) align with these observations, suggesting potential link between magnesium homeostasis and Klotho-related aging processes, highlighting the importance of maintaining adequate magnesium status for healthy aging. The significance of Klotho in aging biology has been extensively demonstrated through genetic studies, where Klotho overexpression extended lifespan by 30% in mice (61), while its deficiency led to premature aging syndromes (62). The Klotho protein, known for its anti-aging properties, has been shown to regulate various physiological processes, including calcium-phosphate homeostasis, oxidative stress resistance, and fibroblast growth factor (FGF) signaling. The work of Kuro-o et al. established Klotho as a critical regulator of aging, with Klotho-deficient mice exhibiting a premature aging phenotype (19). Subsequent research has demonstrated that Klotho’s anti-aging effects extend beyond its original characterization, with studies showing its crucial role in maintaining tissue homeostasis (63) and protecting against age-related organ dysfunction (64). Furthermore, population-based studies have shown that higher circulating Klotho levels are associated with risk of mortality (65) and better preservation of physical function in older adults (66). Our results, showing lower Klotho levels in individuals with higher MDS, suggest that magnesium depletion may contribute to a pro-aging environment by suppressing Klotho expression. This association is particularly noteworthy given that Klotho has been shown to regulate mineral metabolism and influence cellular sensitivity to oxidative stress (21), processes that are also critically dependent on magnesium status (67). This relationship was particularly strong in the high MDS group, indicating that severe magnesium depletion may have a more pronounced effect on Klotho levels and, potentially, on aging processes. These findings have important clinical implications, suggesting the need for regular monitoring of both MDS and Klotho levels in high-risk populations. The clinical relevance of this relationship is underscored by studies demonstrating that interventions aimed at increasing Klotho levels can ameliorate age-related pathologies (68) and improve health outcomes in various chronic conditions (64). The MDS-Klotho axis represents a promising target for early intervention in age-related diseases, potentially offering new approaches to prevent accelerated aging. Future research directions include investigating therapeutic strategies targeting this axis and developing preventive interventions for high-risk populations, which could significantly impact the management of age-related diseases.

The relationship between age, MDS, and Klotho levels deserves special attention in our study. Our findings revealed that participants in the High MDS group were significantly older (mean age 65.13 years) compared to those in the Middle (60.78 years) and Low MDS groups (53.89 years). This age distribution pattern aligns with previous research showing that older adults are more susceptible to magnesium deficiency due to various factors, including reduced intestinal magnesium absorption, increased urinary magnesium excretion, and decreased dietary magnesium intake (69). Notably, our subgroup analyses demonstrated that the association between MDS and serum Klotho levels varied across different age groups, with the strongest and most significant association observed in the 50–59 age group. This age-specific pattern is particularly interesting given that both Klotho levels and magnesium status decline with age (66). The robust association in middle-aged adults (50–59 years) suggests that this might be a critical period where the interaction between magnesium status and Klotho function is most pronounced, potentially representing a key window for preventive interventions. The somewhat attenuated association in older age groups (≥60 years) might reflect the complex interplay of multiple age-related factors affecting both magnesium homeostasis and Klotho expression, including changes in hormone levels, inflammatory status, and renal function. These findings highlight the importance of considering age-specific approaches when developing strategies to maintain optimal magnesium status and Klotho levels, particularly during middle age when interventions might be most effective.

Beyond age-related patterns, the results from our subgroup analysis shed light on specific population groups that may be more vulnerable to the effects of magnesium depletion on Klotho levels. Notably, the association between high MDS and low serum Klotho was particularly pronounced in individuals with a BMI <25, current smokers, and those with low income. These findings suggest that certain lifestyle factors, such as smoking and low socioeconomic status, may exacerbate the detrimental effects of magnesium depletion on Klotho and overall health. Smoking has long been associated with increased oxidative stress and inflammation, which could potentiate the negative effects of magnesium deficiency on Klotho expression. Similarly, low-income individuals may have limited access to magnesium-rich foods, increasing their risk of magnesium deficiency and its associated health consequences. These subgroup-specific findings align with prior research indicating that lifestyle and demographic factors can significantly influence magnesium status and its effects on health outcomes. For instance, a study by Mohammed et al. found that smokers had a significantly higher risk of hypomagnesemia compared to non-smokers, with 24.4% of smokers exhibiting low magnesium levels. This study highlighted a 6.7-fold increased risk of hypomagnesemia in smokers (70). Likewise, individuals with lower socioeconomic status may experience higher rates of magnesium deficiency due to poorer dietary quality and limited access to healthcare (71).

The practical applications of MDS in clinical practice and public health deserve particular attention. As a comprehensive assessment tool, MDS offers distinct advantages over traditional serum magnesium measurements for identifying individuals at risk of aging-related conditions. Unlike serum magnesium measurement alone, MDS provides a more comprehensive evaluation by incorporating multiple clinical risk factors. This makes it valuable as: (1) an initial screening tool before more costly testing, (2) a complement to existing magnesium markers, and (3) a monitoring tool for high-risk patients. The components of MDS are readily available in routine clinical settings, making it a cost-effective and easily implementable screening tool. Our findings suggest that MDS could be particularly valuable for identifying high-risk individuals who might benefit from early interventions, especially among vulnerable populations such as smokers, individuals with low BMI, and those with low income.

From a public health perspective, MDS could be integrated into preventive health programs for risk stratification and targeted interventions. Healthcare providers could use MDS scores to prioritize individuals for lifestyle interventions, nutritional counseling, or more frequent health screenings. Given that MDS incorporates modifiable risk factors such as medication use and alcohol consumption, it could also serve as an educational tool to help patients understand and modify their risk factors for magnesium depletion and accelerated aging. Future research should focus on validating MDS against other established magnesium markers (such as ionized magnesium and intracellular magnesium) and evaluating its predictive value for specific clinical outcomes. The relatively simple nature of MDS calculation makes it suitable for large-scale screening programs and population health management, potentially improving the efficiency of resource allocation in healthcare systems.

Our study has several strengths. This is the first large national study to investigate the association between MDS and serum Klotho levels based on the well-designed NHANES data. In this study, the NHANES sampling weights were fully considered and the analytical instructions were followed. In addition, five cycles (2007–2016) were combined to improve the sampled cohorts and make the results more stable and reliable. Furthermore, MDS rather than serum magnesium was used in our study, which is more reflective of the physiological state of magnesium. The use of this comprehensive score provides a more holistic assessment of magnesium status compared to single biomarkers. However, some limitations should be noted in this study. First, the cross-sectional nature of the study precludes causal inferences about the relationship between magnesium depletion and serum Klotho levels. Longitudinal studies are needed to establish temporality and potential causal relationships. Second, while the MDS provides a more comprehensive assessment of magnesium status than single biomarkers, it is still an indirect measure and may not fully capture intracellular magnesium levels or total body magnesium status. Moreover, magnesium homeostasis is influenced by multiple dietary factors, including protein and fiber intake, which can affect its absorption in the digestive tract. While our study adjusted for total magnesium intake, we did not analyze the specific dietary patterns or nutrients that might influence magnesium absorption. Third, our assessment of kidney function was limited to eGFR measurements without albuminuria data, preventing comprehensive CKD staging. Given the complex and often contradictory evidence regarding Klotho’s role in kidney disease, this limitation may affect the interpretation of our findings. Fourth, while we adjusted for a wide range of potential confounders, residual confounding by unmeasured factors cannot be ruled out. Finally, our study population was limited to US adults aged 40–79 years, and the findings may not be generalizable to younger populations or those from other geographic regions with different dietary patterns and environmental exposures. Future studies should incorporate both eGFR and albuminuria measurements for proper CKD staging, which would help clarify how kidney dysfunction modifies the magnesium-Klotho relationship. Additionally, more direct measures of magnesium status, such as intracellular magnesium concentrations or magnesium loading tests, combined with comprehensive dietary assessments including protein and fiber intake patterns, could provide additional insights.

## 5 Conclusion

Our study provides novel evidence of an inverse association between magnesium depletion, as measured by MDS, and serum Klotho levels in a large, representative sample of US adults. These findings highlight the potential importance of magnesium homeostasis in maintaining adequate Klotho levels, which may have significant implications for healthy aging and age-related diseases. The consistency of this relationship across various subgroups underscores its robustness and potential public health significance. While further research is needed to establish causality and elucidate underlying mechanisms, our results suggest that maintaining adequate magnesium status could be a promising strategy for promoting healthy aging through the modulation of Klotho levels. As the global population continues to age, understanding and leveraging the relationship between magnesium status and Klotho expression may contribute to the development of effective interventions to promote healthier aging and reduce the burden of age-related diseases.

## Data Availability

The original contributions presented in the study are included in the article/Supplementary material, further inquiries can be directed to the corresponding author.

## Footnotes

^1^ https://wwwn.cdc.gov/nchs/nhanes/
