# Supplementary material for: Association of magnesium depletion score with serum anti-aging protein Klotho in the middle-aged and older populations
Source: Front Nutr. 2025 Mar 27;12:1518268. doi: 10.3389/fnut.2025.1518268 (PMC11984463; doi:10.3389/fnut.2025.1518268)
Supplement: Supplementary file 1 [file Table_1.docx]

**Association of magnesium depletion score with serum anti-aging protein klotho in the middle-aged and older populations**

**Table S1**. Associations between magnesium depletion score (MDS) and serum klotho when adjusted for calcium level.

|  | β (95% CI) | |
| --- | --- | --- |
|  | Model 1 | Model 2 |
| MDS | -40.56(-48.18,-32.93) | -41.59(-49.25,-33.92) |
| MDS group |  |  |
| Low | Reference | Reference |
| Middle | -58.83( -77.61,-40.04) | -60.61( -79.44,-41.77) |
| High | -109.48(-134.21,-84.76) | -111.26(-135.83,-86.69) |
| *P* for trend | <0.0001 | <0.0001 |

Model 1: unadjusted;

Model 2: adjusted for calcium level.

**Table S2**. Sensitivity analysis of the associations between magnesium depletion score (MDS) and serum klotho using alternative MDS grouping.

|  | β (95% CI) | | | |
| --- | --- | --- | --- | --- |
|  | Model 1 | Model 2 | Model 3 | Model 4 |
| MDS group |  |  |  |  |
| < 3 | Reference | Reference | Reference | Reference |
| ≥ 3 | -96.29(-120.37,-72.22) | -85.45(-110.06,-60.85) | -83.3(-108.17,-58.43) | -52.6(-101.02, -4.19) |
| P value | <0.0001 | <0.0001 | <0.0001 | 0.03 |

Model 1: unadjusted;

Model 2: adjusted for age, sex, race;

Model 3: adjusted for all the factors in Model 2 and education, marital status, PIR, smoking status, drinking status, BMI, WC, SBP, DBP.

Model 4: adjusted for all the factors in Model 3 and TG, TC, HDL, energy intake.

Abbreviations: CI, confidence interval; MDS, magnesium depletion score; PIR, family poverty income ratio; BMI, body mass index; WC, waist circumference; SBP, systolic blood pressure; DBP, diastolic blood pressure; TG, total triglyceride; TC, total cholesterol; HDL, high-density lipoprotein cholesterol.

**Table S3**. Associations between magnesium depletion score (MDS) and serum klotho when adjusted for magnesium intake.

|  | β (95% CI) | |
| --- | --- | --- |
|  | Model 1 | Model 2 |
| MDS | -40.56(-48.18,-32.93) | -40.73(-48.37,-33.09) |
| MDS group |  |  |
| Low | Reference | Reference |
| Middle | -58.83( -77.61,-40.04) | -59.02( -77.09,-40.96) |
| High | -109.48(-134.21,-84.76) | -109.66(-135.17,-84.15) |
| *P* for trend | <0.0001 | <0.0001 |

Model 1: unadjusted;

Model 2: adjusted for magnesium intake.
